# A highly contiguous nuclear genome assembly of the mandarinfish *Synchiropus splendidus* (Syngnathiformes: Callionymidae)

**DOI:** 10.1093/g3journal/jkab306

**Published:** 2021-10-18

**Authors:** Martin Stervander, William A Cresko

**Affiliations:** Institute of Ecology and Evolution, University of Oregon, Eugene, OR 97403-5289, USA

**Keywords:** mandarin dragonet, barcoded linked-reads, synthetic long-reads, 10x Genomics, Illumina sequencing, syngnathids

## Abstract

The fish order Syngnathiformes has been referred to as a collection of misfit fishes, comprising commercially important fish such as red mullets as well as the highly diverse seahorses, pipefishes, and seadragons—the well-known family Syngnathidae, with their unique adaptations including male pregnancy. Another ornate member of this order is the species mandarinfish. No less than two types of chromatophores have been discovered in the spectacularly colored mandarinfish: the cyanophore (producing blue color) and the dichromatic cyano-erythrophore (producing blue and red). The phylogenetic position of mandarinfish in Syngnathiformes, and their promise of additional genetic discoveries beyond the chromatophores, made mandarinfish an appealing target for whole-genome sequencing. We used linked sequences to create synthetic long reads, producing a highly contiguous genome assembly for the mandarinfish. The genome assembly comprises 483 Mbp (longest scaffold 29 Mbp), has an N50 of 12 Mbp, and an L50 of 14 scaffolds. The assembly completeness is also high, with 92.6% complete, 4.4% fragmented, and 2.9% missing out of 4584 BUSCO genes found in ray-finned fishes. Outside the family Syngnathidae, the mandarinfish represents one of the most contiguous syngnathiform genome assemblies to date. The mandarinfish genomic resource will likely serve as a high-quality outgroup to syngnathid fish, and furthermore for research on the genomic underpinnings of the evolution of novel pigmentation.

## Introduction

**Figure 1:**
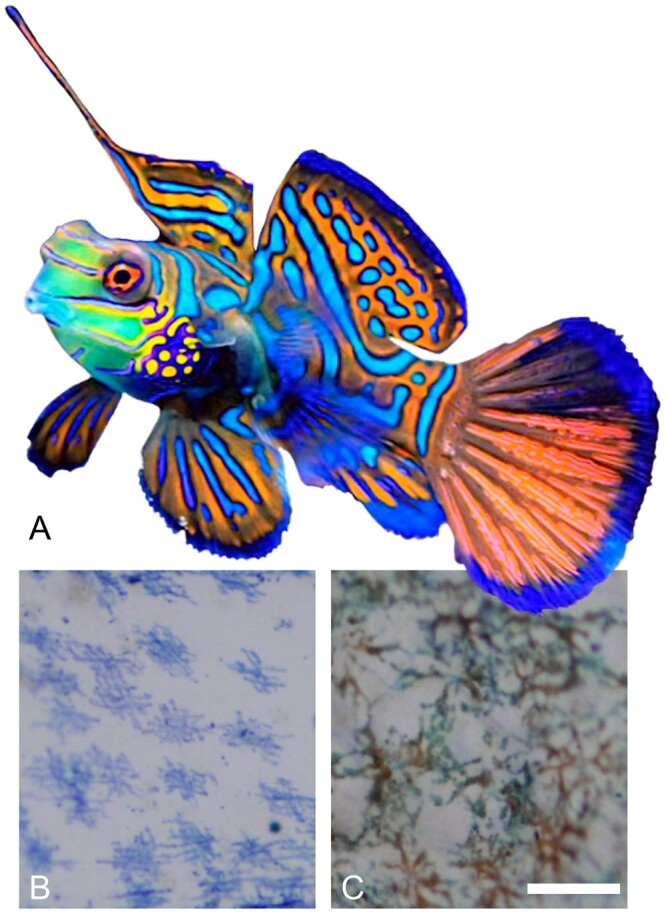
Two chromatophore types have been discovered in the mandarinfish *Synchiropus splendidus*. (A) A male mandarinfish (image by user Ultimatemonty at FavPng, https://favpng.com/) demonstrating the vibrant colors of the species. (B, C) Light microscopy images of mandarinfish pectoral fin tissue, adapted from Goda *et al.* (2013) with permission. The micrographs depict the two novel chromatophore types discovered in the mandarinfish: (B) dermal cyanophores and (C) dichromatic chromatophores, situated around the edge of the blue regions, displaying producing both blue and red either separately or together in the cytoplasm. Length bars correspond to 50 μm.

The mandarinfish *Synchiropus splendidus* (Herre 1927), also known as a mandarin dragonet, is an intensely colored west Pacific species (Figure 1) that is popular in aquarium trade, even though captive breeding is difficult and most specimens therefore are wild-caught (Sadovy *et al.* 2001). Aside from the commercial interest in the species, the evolution of such vibrant coloration has captured the eye and attention of biologists. Studies of mandarinfish coloration have been the source for the discovery of two types of chromatophores. While blue colors in animals are generally structural, the mandarinfish has cyanophores producing a strong blue color (Goda and Fujii 1995). More recently a dichromatic chromatophore producing blue and red was described in mandarinfish (Goda *et al.* 2013).

The phylogenetic and taxonomic placement of the dragonet families Draconettidae and Callionymidae—to which the mandarinfish belongs—has long been subject for debate. Morphology-based assessment has traditionally placed dragonets with a phylogenetic affinity to clingfishes (within order Gobiesociformes; *e.g.*, Springer and Johson 2004) or independently comprising the order Callionymiformes (*e.g.*, Nelson *et al.* 2016). Early molecular studies based on a few, predominantly mitochondrial sequence markers also resulted in wildly different phylogenetic placements of dragonets (Chen *et al.* 2003; Smith and Wheeler 2006). However, molecular studies of larger genetic material have demonstrated that dragonets form a monophyletic clade with the morphologically distant families Syngnathidae, Solenostomidae, Aulostomidae, Fistulariidae, Centriscidae, Dactylopteridae, Mullidae, and Pegasidae, albeit with varying internal arrangements (Kawahara *et al.* 2008; Betancur-R *et al.* 2013, 2017; Near *et al.* 2013; Sanciangco *et al.* 2016; Longo *et al.* 2017; Alfaro *et al.* 2018; Hughes *et al.* 2018).

**Figure 2:**
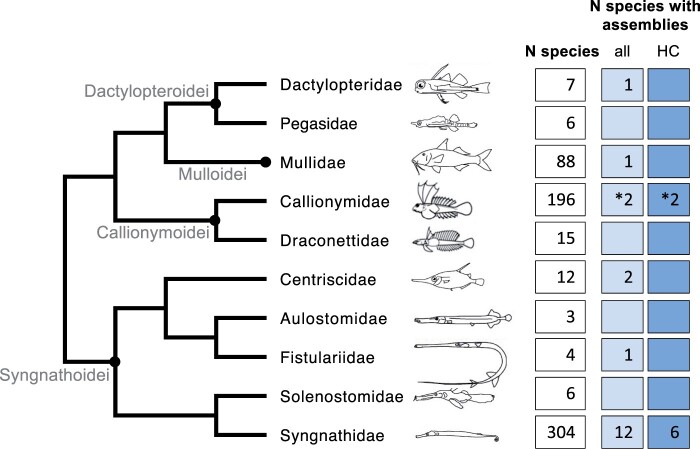
Families of order Syngnathiformes (entire phylogeny), ordered in suborders (grey text, black circles at nodes) according to Betancur-R *et al.* (2017). In boxes are the number of species according to Fishbase (Froese and Pauly 2019), the number of species with available nuclear *de novo* genome assemblies, and the number of assemblies which are of higher contiguity (HC; scaffold N50 > 0.5 Mbp). *Signifies the addition of one species from this study. Sketches from Longo *et al.* (2017) and Song *et al.* (2014).

The clade composed of the aforementioned families makes up order Syngnathiformes (sensu Betancur-R *et al.* 2017; Hughes *et al.* 2018), in which genome assemblies have been available for suborders Syngnathoidei, Dactylopteroidei, and Mulloidei (sensu Betancur-R *et al.* 2017; cf. Figure 2), and the first genome assembly for suborder Callionymoidei was only recently published (Winter *et al.* 2020). Whereas several high-quality assemblies within family Syngnathidae, *i.e.*, seahorses, pipefishes, and seadragons have been published in the last few years (Lin *et al.* 2016; Small *et al.* 2016; Vertebrate Genomes Project 2019; Roth *et al.* 2020; Zhang *et al.* 2020; Li *et al.* 2021), nonsyngnathid syngnathiform species are generally represented by relatively fragmented assemblies (scaffold N50 17–116 Kbp) of low coverage (29–58×; Roth *et al.* 2020). An exception with intermediate contiguity and coverage is the striped red mullet *Mullus surmuletus* (scaffold N50 483 Kbp, 73× coverage; Fietz *et al.* 2020), and importantly, the recent chromosome-level genome assembly of the common dragonet *Callionymus lyra* that was produced by Winter *et al.* (2020), based on MinION sequencing. The creation of several additional, high-quality reference genomes from across Syngnathiformes is therefore important in general for the genetic analysis of the amazing phenotypic diversity in this clade. To meet this research need, and to create a key resource for the genetic analysis of pigment evolution in dragonets, we present in this study a highly contiguous nuclear genome assembly of a species within suborder Callionymoidei, the mandarinfish.

## Materials and methods

### Sample acquisition, tissue collection, DNA extraction, and sequencing

We purchased an adult male mandarinfish *S. splendidus*, that had been collected close to the Kapal and Kahyangan Islands north of Java, Indonesia, at approximately 6°02′10″S 106°44′05″E, through a commercial aquarium fish trader (Blue Zoo Aquatics, Gardena, CA, USA). Upon receipt of the fish on March 20, 2019, we euthanized it using 0.0168% tricaine methanesulfonate (MS-222), immediately dissected it, and flash froze tissues separately in liquid nitrogen, followed by storage at –80°C. All vertebrate handling and euthanization followed approved, IACUC-regulated protocols (University of Oregon #17-05).

We extracted high molecular weight DNA using a prototype Nanobind Tissue Big DNA Kit (Circulomics, Baltimore, MD, USA), modified as follows: We mechanically homogenized 15 mg liver tissue within a tissueTUBE TT1 (Covaris, Woburn, MA, USA) by chilling the tissue for ∼5 s in liquid nitrogen, then immediately crushing it with a pre-chilled steel hammer and anvil. The homogenate was then used for extraction according to the manufacturer’s instructions, with elution in 100 μl elution buffer EB. The DNA was quantified using a Qubit dsDNA BR Assay on a Qubit 2.0 Fluorometer (Thermo Fisher, Waltham, MA, USA), purity was checked on a Nanodrop 2000 (Thermo Fisher, Waltham, MA, USA), and DNA size distribution was determined with a HS Large Fragment 50Kb Kit on a Fragment Analyzer (Agilent, Santa Clara, CA, USA).

We ran 3.5 μg genomic DNA on a BluePippin (Sage Science, Beverly, MA, USA), collecting fragments ≥45 Kbp, which were used to construct a whole-genome linked-reads library with a Chromium Genome Reagent Kit (10x Genomics, Pleasanton, CA, USA) following the manufacturer’s instructions. The synthetic long-read library was checked with an HS NGS Fragment Kit (1–6000 bp) on a Fragment Analyzer (Agilent, Santa Clara, CA, USA), and then loaded on half a lane on a Hiseq 4000 (Illumina, San Diego, CA, USA) in the University of Oregon Genomics & Cell Characterization Core Facility (GC3F) which produced 152 million 150-bp, paired-end reads.

### Genome assembly and assessment

In order to assess the raw data and make a preliminary estimations of the genome size, level of heterozygosity, and read error rate, we trimmed all reads with Trimmomatic v. 0.36 (Bolger *et al.* 2014) using arguments LEADING: 3 TRAILING: 3 SLIDINGWINDOW: 4:15 MINLEN: 36. We also ran kmer analyses at kmer sizes 19–31 bp with Jellyfish v. 1.1.11 (Marçais and Kingsford 2011) on the remaining 125,818,427 read pairs. We then used the generated histo files for genome profiling with Genomescope v. 1 (Vurture *et al.* 2017) at kmer size = 19, 25, and 31 bp.

For the assembly, we followed the recommendations from 10x Genomics and used raw (nontrimmed) reads as input to Supernova v. 2.0.1 (Weisenfeld *et al.* 2017) because the assembly pipeline addresses trimming needs and no advantage has been demonstrated by trimming reads ahead of assembly (10x Genomics 2019). We ran the genome assembly with all 152,197,312 raw read pairs on a single core of Talapas, University of Oregon’s high performance computing environment, with 28 CPU and 114 Mb RAM for 47 h. The result of this assembly was two pseudohaplotypes. Supernova is known to produce duplicate scaffolds (Ozerov *et al.* 2020), and among the 10,652 scaffolds (referred to as contigs by 10x Genomics), 1441 were identified as duplicates and therefore removed with seqkit rmdup (Zou *et al.* 2016). One scaffold was removed because it consisted of Ns only. We also detected seven instances where Supernova failed to remove one of two Illumina adaptor sequences at the end of a scaffold (one instance) or within scaffolds between true sequence and runs of Ns (six instances). While contamination of adaptor sequence usually indicates a risk of mis-assembly, because reads may have been mistakenly aligned specifically based on matching adaptor sequence, this risk should be low based on the location of those adaptor sequence remnants and the DNA molecule-specific barcoding.

We evaluated the de-duplicated pseudohaplotype 1 of the assembly using Quast v. 4.6.0 (Gurevich *et al.* 2013), and assessed assembly completeness by searching for near-universal single-copy orthologous genes in the ray-finned fish (actinopterygii_odb9) and vertebrate (vertebrata_odb10) ortholog sets with BUSCO v. 3.0.2 (Waterhouse *et al.* 2018).

### Repeat content analyses

We ran RepeatModeler v. 1.0.11 (Smit and Hubley 2014) using the NCBI engine and combined the custom repeat library with publicly available fish repeats (RepeatMasker queryRepeatDatabase.pl -species Teleostei), which we used with RepeatMasker v. 4.0.9 (Smit *et al.* 2014) with databases Dfam_3.0, RepBase-20170127 and arguments -norna -xsmall.

## Results and discussion

Using the prototype Nanobind Tissue Big DNA Kit, the yield was 11.69 μg DNA extracted from 15 mg liver tissue, with absorbance ratios at 260/230 nm of 2.13, and at 260/280 nm 1.83. The DNA produced was of high molecular weight, with a mode size of 50,933 bp. The size distribution contained 51.6% of the DNA among fragments ≥40 Kbp, 37.6%, ≥50 Kbp, 22.5% ≥40 Kbp, and 9.8% ≥75 Kbp, which allowed for size selection of ≥45 Kbp.

### Genome assembly and repeat content

The preliminary kmer analyses with Genomescope estimated a genome size of 481–492 Mbp a heterozygosity level of 1.20%–1.31% (Table 1). The final assembly had an effective coverage of 59× (raw coverage of 81×), was highly contiguous, and comprised 483 Mbp, with N50 > 12 Mbp and L50 of 14 scaffolds (Table 2).

**Table 1:** Estimations of nuclear genome size and heterozygosity for the mandarinfish *Synchiropus splendidus*, based on kmer analyses in Genomescope of 126 million trimmed read pairs

Kmer length	Genome size (Mbp)	Heterozygosity (%)
19	481	1.31
25	488	1.26
31	492	1.20

**Table 2:** Summary statistics for the mandarinfish *Synchiropus splendidus* nuclear genome assembly fSynSpl_1.0

Assembly statistic	fSynSpl_1.0
Number of scaffolds	9,210
Total length	482.93 Mbp
Largest scaffold	29.25 Mbp
N50	12.19 Mbp
N75	5.85 Mbp
L50	14
L75	27
GC content	43.79%
Number of Ns per Kbp	36.21

The assembly completeness was relatively high as supported by the finding that only 2.9% of 4584 Actinopterygii BUSCO genes were not recovered and 4.4% were fragmented. The remaining 92.6% of BUSCO genes were complete in our assembly, with similar figures for the Vertebrata gene set (Table 3). Total repeat content of the genome was 17.6%, comprising predominantly DNA elements (5.87%), LINEs (2.47%), and simple repeats (1.39%; Table 4). These data are comparable to other syngnathiform assemblies (Lin *et al.* 2016, 2017; Small *et al.* 2016; Roth *et al.* 2020; Zhang *et al.* 2020), where total repeat content (albeit not calculated identically between studies) ranges between 11.5% and 68.5% of the assembly size. The repeat content largely determines assembly size (linear regression: *b* = 7.52, *t*_1,16_ = 5.21, *r*^2^ = 0.63, *P* < 0.0001). A phylogenetic signal of repeat content exists because species of the subfamily Nerophinae (Syngnathoidei: Syngnathidae; *n* = 4 assemblies) have a higher ratio of repeat: nonrepat content (1.00–2.17) compared to species within the other syngnathid subfamily Syngnathinae (0.18–0.49; *n* = 7), other families within Syngnathoidei (Centriscidae and Fistulariidae; 0.13–0.30; *n* = 3), and representatives of suborders Mulloidei (0.20; *n* = 1), Dactylopteroidei (0.32; *n* = 1), and Callinymoidei (0.21–0.37; *n* = 2; this study).

**Table 3:** BUSCO assessment of the mandarinfish *Synchiropus splendidus* nuclear genome assembly (fSynSpl_1.0) completeness through searching for single-copy orthologs from the Vertebrata and Actinopterygii datasets

BUSCOs	Vertebrata	Actinopterygii
Complete	3,074 (91.7%)	4,247 (92.6%)
single copy	3,045 (90.8%)	4,127 (90.0%)
duplicated	29 (0.9%)	120 (2.6%)
Fragmented	145 (4.3%)	202 (4.4%)
Missing	135 (4.0%)	135 (2.9%)
Total	3,354	4,584

**Table 4:** Repeat contents of the mandarinfish *Synchiropus splendidus* nuclear genome assembly (fSynSpl_1.0), determined with RepeatMasker, using a custom assembly-specific repeat library and publicly available repeats in Teleostei

Repeat type	*N* elements	Σ sequence length (bp)	Proportion of assembly (%)
Total interspersed repeats		76,558,225	15.85
SINEs	3,898	376,741	0.08
ALUs	0	0	0.00
MIRs	180	20,908	0.00
LINEs	57,871	11,932,669	2.47
LINE1	2,822	772,252	0.16
LINE2	5,219	884,904	0.18
L3/CR1	0	0	0.00
LTR	22,877	5,559,049	1.15
ERVL	139	200,533	0.04
ERVL-MaLRs	0	0	0.00
ERV classI	1,630	501,062	0.10
ERV classII	110	41,130	0.01
DNA elements	151,460	28,367,333	5.87
hAT-Charlie	30,384	5,308,847	1.10
TcMar-Tigger	10,929	3,438,056	0.71
Unclassified	199,626	30,322,433	6.28
Satellites	1,161	179,831	0.04
Simple repeats	142,303	6,729,931	1.39
Low complexity	16,696	849,897	0.18

Since the input DNA for the synthetic long-read library was primarily long fragments (≥45 Kbp), the 16,430 bp mitochondrion (Song *et al.* 2014) was not assembled. The absence of the mtDNA genome was confirmed by BLAST for *cytochrome b*, *COI*, *ND1*, and *ND4*, which resulted in short (27–236 bp) best hits at low similarity (pairwise identity 75%–93%, *e*-values at 10^−1^–10^−60^).

### Synthetic long-read sequencing

The development of high-throughput sequencing continues to be rapid, with decreasing user costs (Goodwin *et al.* 2016). Long-read technologies, such as PacBio and Oxford Nanopore, can produce chromosome-level assemblies in combination with chromatin conformation capture techniques such as Hi-C (*e.g.*, Ge *et al.* 2019; Low *et al.* 2019; Pettersson *et al.* 2019; Kirubakaran *et al.* 2020). However, sequencing costs as well as the amount of DNA required for long-read library preparation may still pose a barrier. Here, *synthetic long reads* based on linked short-read sequencing may come to play an important role, as the required DNA input is very low (0.1–1.25 ng) and the sequencing is carried out on regular short-read platforms, at much lower costs. Since our sequencing of the mandarinfish, 10x Genomics has discontinued their linked-reads genomic library kits, but alternative synthetic long-read methodologies based on similar strategies have been launched with TELL-seq (Chen *et al.* 2020) and stLFR (Wang *et al.* 2019). Linked-read sequencing approaches will continue to be a useful NGS arrow in a genomicist’s quiver.

### The mandarinfish assembly as genomic resource for studies of vertebrate pigmentation

As many a seasoned ichthyologist or an aquarium hobbyist can attest, the body coloration of mandarinfish is vivid. This striking overall coloration patterning is matched by a striking cell biology discovery. Of less than a dozen chromatophores yet known from all species of fish (Sköld *et al.* 2016), two were discovered in the mandarinfish (Goda and Fujii 1995; Goda *et al.* 2013). The pigmentation patterns created by chromatophores have a complex genetic background (Irion *et al.* 2016; Cal *et al.* 2017). While most studies of pigmentation development and evolution have used more traditional models (Kronforst *et al.* 2012), including mice (Hoekstra *et al.* 2006) and zebrafish *Danio rerio* (Irion *et al.* 2016; Patterson and Parichy 2019), expanding the scope to the relatives of model species (Spiewak *et al.* 2018; McCluskey *et al.* 2021), as well as to diverse clades of organisms such as cichlids (Albertson *et al.* 2014) and other teleost fish (Parichy 2021), is yielding even more discoveries. The present assembly of the mandarinfish offers an excellent resource to explore the genetic basis of pigmentation in cyanophores (Goda and Fujii 1995) and the unique dichromatic cyano-erythrophores (Goda *et al.* 2013), in combination with overall RNA sequencing and genome annotation, as well as single-cell RNA sequencing of chromatophore cells from the mandarinfish skin.

In addition, the highly contiguous mandarinfish genome assembly will, together with the common dragonet assembly (Winter *et al.* 2020), be an important comparative genomics resource as an outgroup to the highly derived family Syngnathidae, known for its unique vertebrate innovation of male pregnancy, through the brooding of eggs and young in a body pouch. The evolution and the genomic basis of this trait has attracted much recent attention (Lin *et al.* 2016; Small *et al.* 2016; Roth *et al.* 2020; Zhang *et al.* 2020), but other remarkable adaptations include a craniofacial morphology allowing specialized pivot suction feeding through a toothless, tubular mouth; hard body armor; bony spines; prehensile tails; elongated body plan and loss of fins; and camouflage through elaborate appendages (*e.g.*, Ahnesjö and Craig 2011; Lin *et al.* 2016; Small *et al.* 2016; Li *et al.* 2021). Similar to family Syngnathidae, other families within suborder Syngnathoidei display elongated snouts (Solenostomidae, Fistulariidae, Aulostomidae, and Centriscidae; Figure 2) and body plans (Solenostomidae, Fistulariidae, and Aulostomidae). The mandarinfish will therefore also represent a beautiful (and useful) outgroup to suborder Syngnathoidei.

## Data availability

The data underlying this article can be accessed with accession number JAFFPX000000000 from the GenBank Assembly Database at https://www.ncbi.nlm.nih.gov/assembly, and with accession number SRR12233697 from the GenBank Sequence Read Archive at https://www.ncbi.nlm.nih.gov/sra, both connected to accession number PRJNA646594 in the GenBank BioProject Database at https://www.ncbi.nlm.nih.gov/bioproject.
